# Genetic characterization of measles viruses isolated in Turkey during 2000 and 2001

**DOI:** 10.1186/1743-422X-2-58

**Published:** 2005-07-19

**Authors:** Gulay Korukluoglu, Stephanie Liffick, Dalya Guris, Fumio Kobune, Paul A Rota, William J Bellini, Ali Ceylan, Meliksah Ertem

**Affiliations:** 1 National Measles/Rubella Laboratory, Refik Saydam National Hygiene Center, Ankara, Turkey; 2 Division of Viral and Rickettsial Diseases, Centers for Disease Control and Prevention, Atlanta, Georgia, USA; 3 National Immunization Program, Centers for Disease Control and Prevention, Atlanta, Georgia, USA; 4 Biomedical Sciences Association, Tokyo, Japan; 5 Department of Public Health, Dicle University School of Medicine, Diyarbakir, Turkey

## Abstract

**Background:**

Molecular epidemiologic studies have made significant contributions to measles surveillance activities by helping to identify source and transmission pathways of the virus. This report describes the genetic characterization of wild-type measles viruses isolated in Turkey in 2000 and 2001.

**Results:**

Wild-type measles viruses were isolated from 24 cases from five provinces in Turkey during 2001. The viruses were analyzed using the standard genotyping protocols. All isolates were classified as genotype D6, the same genotype that was identified in Turkey in previous outbreaks during 1998.

**Conclusion:**

Turkey has begun implementation of a national program to eliminate measles by 2010. Therefore, this baseline genotype data will provide a means to monitor the success of the elimination program.

## Background

Measles virus (MV), an enveloped virus with a single-stranded, negative sense RNA genome, is a member of the genus *Morbillivirus *within the family *Paramyxoviridae*. MV is highly contagious and causes a disease characterized by high fever, cough, coryza, conjunctivitis and appearance of a maculopapular rash [1]. In many parts of the world, vaccination programs have controlled measles. However, despite the tremendous progress of global measles control, MV is still responsible for the deaths of approximately 700,000 thousand children each year, mostly in developing countries [2]. Measles remains the most common of vaccine-preventable childhood mortality.

Although MV is considered to be monotypic, genetic variability exists among wild type strains [3]. Genetic characterization of wild-type MVs is based on sequence analysis of a hypervariable region (450 nt) of the nucleoprotein (N) gene and the full-length hemagglutinin (H) gene. A standard nomenclature and analysis protocol for describing the genetic characteristics of wild-type MVs was established by the World Health Organization (WHO) [4-7]. WHO recommends that genetic analysis of MV isolates should be conducted during all phases of measles control. Genetic analysis of wild-type MVs has provided an increasingly comprehensive picture of the worldwide distribution of MV genotypes [8]. Molecular epidemiologic studies can help to measure transmission pathways and to clarify epidemiological links during outbreaks. Virologic surveillance can also help to measure the success of measles vaccination programs by documenting the interruption of transmission of the endemic viral genotype(s) [9,10].

In 2001, Turkey experienced a large measles epidemic and the number of reported measles cases was over 30,000 [11]. From October 2000 to August 2001, we isolated MVs from measles cases in five different provinces of Turkey. Since Turkey has recently initiated a program to eliminate measles, this report provides important baseline data that will allow future molecular epidemiologic studies to help measure the success of this program.

## Results and Discussion

**Figure 1:**
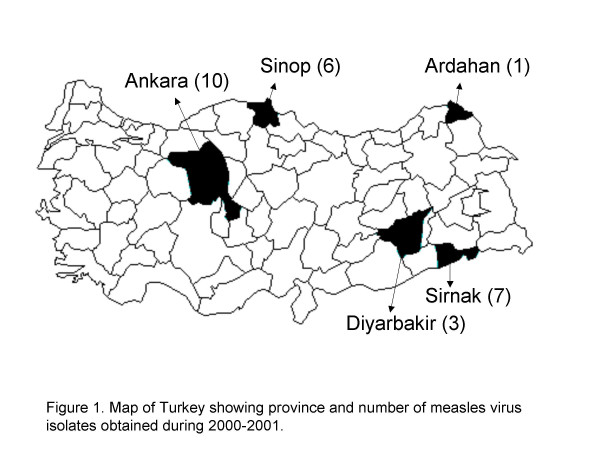
Map of Turkey showing province and number of measles virus isolates obtained during 2000–2001.

With the exception of one specimen that was collected in October 2000, the remaining specimens were collected between February and August in 2001 (Table 1). MV isolates were obtained from 24 specimens collected from widely dispersed areas of Turkey, including the provinces of Ankara, Sinop, Diyarbakir, Sirnak, and Ardahan (Figure 1, Table 1). Measles specific IgM antibody was detected in serum samples from 16 of 20 cases, while serologic results were not available for 4 cases. The serum samples from 3 of the 4 IgM negative cases were taken 2 days after rash onset when the sensitivity of IgM detection is low.

**Table 1:** Epidemiological and serological information on measles virus isolates from Turkey.

**WHO Name [Genotype]**	**Age**	**Measles IgM**	**Date of after rash**	**Cell lines used for isolation**	**Type of specimen**	**Province**	**Epi-link**
MVi/Ankara.TUR/38.00 [D6]	7 y	positive	3	B95 a	urine	Ankara	sporadic
MVi/Ankara.TUR/05.01 [D6]	17 y	negative	2	B95 a	urine	Ankara	sporadic
MVi/Ankara.TUR/06.01-1 [D6]	24 y	negative	2	B95 a	urine	Ankara	sporadic
MVi/Ankara.TUR/06.01-2 [D6]	21 y	negative	2	B95 a	urine	Ankara	epidemic
MVi/Ankara.TUR/07.01 [D6]	2.5 y	?	?	B95 a	urine	Ankara	sporadic
MVi/Sinop.TUR/11.01-1 [D6]	13 y	positive	4	B95 a	urine	Sinop	epidemic
MVi/Sinop.TUR/11.01-2 [D6]	13 y	positive	5	B95 a	urine	Sinop	epidemic
MVi/Sinop.TUR/11.01-3 [D6]	13 y	positive	4	B95 a	throat swab	Sinop	epidemic
MVi/Sinop.TUR/11.01-4 [D6]	13 y	positive	3	B95 a	urine	Sinop	epidemic
MVi/Sinop.TUR/11.01-5 [D6]	13 y	positive	4	B95 a	urine	Sinop	epidemic
MVi/Sinop.TUR/11.01-6 [D6]	13 y	positive	4	B95 a	throat swab	Sinop	epidemic
MVi/Ankara.TUR/14.01 [D6]	?	positive	?	B95 a	nasal swab	Ankara	sporadic
MVi/Ankara.TUR/19.01-1 [D6]	25 y.	?	3	COBL	urine	Ankara	sporadic
MVi/Ankara.TUR/19.01-2 [D6]	?	?	?	COBL	urine	Ankara	sporadic
MVi/Ardahan.TUR/23.01 [D6]	?	?	?	COBL	urine	Ardahan	epidemic
MVi/Sirnak.TUR/29.01-1 [D6]	3 y.	negative	5	COBL	urine	Şırnak	epidemic
MVi/Sirnak.TUR/29.01-2 [D6]	3 y.	positive	3.	COBL	throat swab	Şırnak	epidemic
MVi/Sirnak.TUR/29.01-4 [D6]	3 y.	positive	7	COBL	urine	Şırnak	epidemic
MVi/Sirnak.TUR/29.01-5 [D6]	4 y.	positive	3	COBL	urine	Şırnak	epidemic
MVi/Sirnak.TUR/29.01-6 [D6]	2 y.	positive	5	COBL	urine	Şırnak	epidemic
MVi/Sirnak.TUR/29.01-7 [D6]	8 mo.	positive	6	COBL	blood	Şırnak	epidemic
MVi/Diyarbakir.TUR/30.01-1 [D6]	7 y.	positive	4	COBL	blood	Diyarbakır	epidemic
MVi/Diyarbakir.TUR/30.01-2 [D6]	7 y.	positive	2	COBL	urine	Diyarbakır	epidemic
MVi/Ankara.TUR/30.01 [D6]	2 y.	positive	?	COBL	urine	Ankara	sporadic

**Figure 2:**
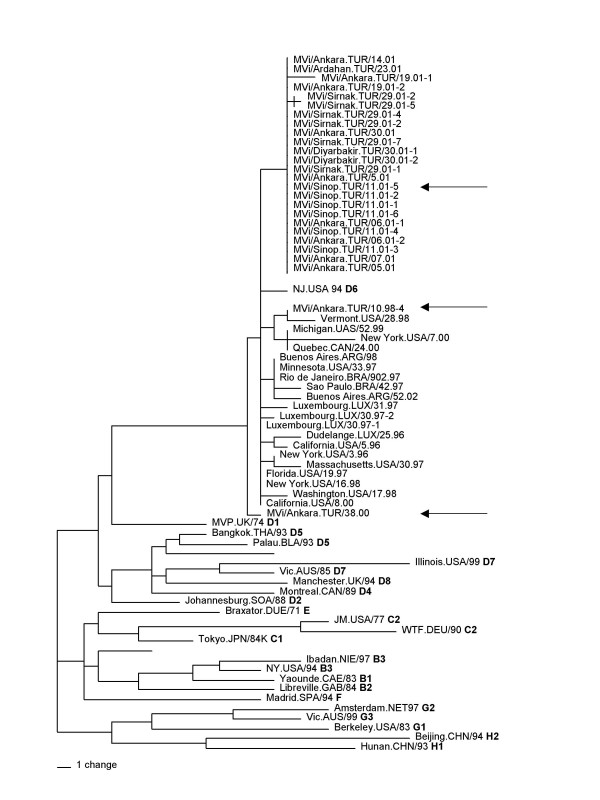
Phylogenetic analysis of the N gene sequences of wild-type MVs isolated in Turkey. Sequences of the Turkish viruses were compared to the sequence of the WHO reference strains (genotype shown in bold). Turkish viruses are indicated by arrows. Sequences of previously described genotype D6 viruses [10, 8–20] are also included in this un-rooted tree.

Comparison of the N gene sequences of the Turkish viruses with the sequences of the current of WHO reference strains showed that all 24 Turkish strains were members of genotype D6 (Figure 2). The sequences of the Turkish viruses were closely related to each other showing no more than 1.3% nucleotide heterogeneity overall. In fact, the N gene sequences of 21 of these MV isolates were identical, though they came from different regions of Turkey. Although the Turkish viruses were clearly in genotype D6, the sequences of the more recently isolated viruses formed a distinct group relative to other genotype D6 viruses recently isolated in Germany, Luxembourg, Brazil and the United States [10,18-20]. However, the nucleotide sequences from the Turkish cluster differed from the sequences of the non-Turkish viruses by no more than 1.1% overall. The sequence of a single isolate from Ankara in 2000, MVi/Ankara.TUR/38.00, and a genotype D6 isolate from the 1998 outbreak, MVi/Ankara/10-98-4 [21], were more closely related to the sequences of the European, and Brazilian genotype D6 viruses than the sequences of the Turkish cluster (Figure 2).

At present, genotype D7 appears to be the most frequently detected genotype in Western European countries; however, D6 genotype is still circulating in some European countries including the Russian Federation [6,19]. Genotype D6 viruses were imported to the United States from various European countries and Brazil on 13 occasions between 1997 and 2000; however, after 2000, only 2 genotype D6 viruses were detected in the United States (Rota, unpublished).

In some parts of Europe, measles is near elimination or has been eliminated, whereas in others measles is still endemic [22]. Despite an active vaccination program, measles has been an endemic disease in Turkey with epidemics occurring every 3–4 years. In 2001, the last epidemic year, over 30.000 cases were reported [11]. The previous epidemic year was 1998, when more than 27,000 cases were reported. The virologic surveillance data suggest that viruses in genotype D6 were responsible for both epidemics and continued to circulate during the inter-epidemic periods.

To reduce measles morbidity and mortality in Turkey, the Ministry of Health launched a National Measles Elimination Program in 2002. In parallel with the strategic plan of the European Regional Office of WHO, the Turkish national plan targets elimination of measles by 2010 [23]. The plan included a "catch-up" vaccination campaign targeting nearly 20 million children between 9 months and 14 years of age to be conducted in two phases during December 2003 and 2005 [24]. The National Measles Plan also includes activities for establishing a laboratory based surveillance system to monitoring the effectiveness of the measles elimination program [25]. In Turkey, sub-national laboratories from seven selected provinces will carry out laboratory-based surveillance, each representing a region of the country. These sub-national laboratories will perform serologic confirmation of suspected measles cases. Clinical specimens collected from laboratory-confirmed cases will be sent to the National Measles and Rubella Laboratory for virus isolation and genotyping.

## Conclusion

Genetic analysis of MVs isolated after the measles vaccination campaigns will help to determine if the circulation of the endemic genotype D6 viruses is interrupted. This analysis would not be possible without the baseline data presented in this report. Turkey is in a unique geographic position to monitor transmission of measles virus between Europe, the Middle East and the rest of Asia. Strengthening virologic surveillance capacity in Turkey will benefit several WHO regions.

## Materials and methods

### Clinical specimens

Urine, nasopharyngeal secretions and blood samples were collected from 24 patients who had acute, febrile maculopapular rash from five different provinces in Turkey. All clinical samples were collected within six days of rash onset and transported to Refik Saydam Hygiene Center, National Measles and Rubella Laboratory in accordance with standard protocols (Table 1). Isolation of MV was performed using the B95a cell line (12) for 12 samples and the COBL cell line (IL-II treated human cord blood cells, 13) for 15 samples. Syncytia formation, the cytopathic effect (CPE) characteristic of MV infection, appeared within 1–7 days. When the CPE was advanced the cultures were harvested and stored at -80°C. All isolates were confirmed as measles by a neutralization test performed by using monospecific rabbit antibody to the H protein.

### Sequence analysis

RNA was extracted from infected cells using the guanidinium acid-phenol technique [14]. The 450 nucleotides corresponding to the COOH-terminal 150 amino acids of the N protein were amplified by using a one-step RT-PCR kit according to manufacturer's protocol (Superscript, Invitrogen). Forward and reverse primers were: 5'GCTATGCCATGGGAGTAGGAGTGG and 5'CTGGCCCTCGGCCTCTCGCAC, respectively. Sequences of the PCR products were derived by automated sequencing with the BigDye terminator VI.I chemistry according to the manufacturer's protocol (Perkin Elmer-Applied Biosystems, Foster City, CA). Sequence reaction product results were analyzed on an automatic sequencer (ABI 3100, Perkin Elmer Applied Biosystems, Foster City, CA). Sequence data were analyzed by using version 10.0 of the Genetics Computer Group Sequence Analysis Software Package [15] and phylogenetic analyses were performed using PHYLIP ver 3.4 [16] and PAUP ver 4.0 [17]. All phenograms were drawn as unrooted trees. Sequence data were deposited in GenBank under accession numbers (AY899306-AY899329).

## List of Abbreviations

MV: measles virus

N: nucleoprotein

COOH- carboxyl

WHO: World Health Organization

## Competing interests

The author(s) declare that they have no competing interests.

## Authors' contributions

GK, FK, AC, ME collected specimens and performed virus isolation and measles IgM assays; GK, FK established COBL cell in the Ankara laboratory; GK, SL, PR performed RT-PCR and sequence analysis; GK, DG, PP, WB analyzed data and prepared draft manuscript. All authors revised manuscript and approved final draft.
